# Prevalence and determinants of diarrhea among under-five children in Ethiopia: A systematic review and meta-analysis

**DOI:** 10.1371/journal.pone.0199684

**Published:** 2018-06-28

**Authors:** Animut Alebel, Cheru Tesema, Belisty Temesgen, Alemu Gebrie, Pammla Petrucka, Getiye Dejenu Kibret

**Affiliations:** 1 College of Health Sciences, Debre Markos University, Debre Markos, Ethiopia; 2 Debre Markos Referral Hospital, Debre Markos, Ethiopia; 3 College of Nursing, University of Saskatchewan, Saskatoon, Canada; 4 School of Life Sciences and Bioengineering, Nelson Mandela African Institute of Science and Technology, Arusha, Tanzania; South Gloucestershire, UNITED KINGDOM

## Abstract

**Background:**

Despite remarkable progress in the reduction of under-five mortality, childhood diarrhea is still the leading cause of mortality and morbidity in this highly susceptible and vulnerable population. In Ethiopia, study findings regarding prevalence and determinants of diarrhea amongst under-five children have been inconsistent. Therefore, this systematic review and meta-analysis estimates the pooled prevalence of diarrhea and its determinants among under-five children in Ethiopia.

**Methods:**

International databases, including PubMed, Web of Science, EMBASE, CINAHL, Google Scholar, Science Direct, and the Cochrane Library, were systematically searched. All identified observational studies reporting the prevalence and determinants of diarrhea among under-five children in Ethiopia were included. Two authors independently extracted all necessary data using a standardized data extraction format. STATA Version 13 statistical software was used. The Cochrane Q test statistics and *I*^*2*^ test were used to assess the heterogeneity of the studies. A random effects model was computed to estimate the pooled prevalence of diarrhea. Moreover, the associations between determinant factors and childhood diarrhea were examined using the random effect model.

**Results:**

After reviewing of 535 studies, 31studies fulfilled the inclusion criteria and were included in the meta-analysis. The findings from the 31 studies revealed that the pooled prevalence of diarrhea among under-five children in Ethiopia was 22% (95%CI: 19, 25%). Subgroup analysis of this study revealed that the highest prevalence was observed in Afar region (27%), followed by Somali and Dire Dawa regions (26%), then Addis Abeba (24%). Lack of maternal education (OR: 2.5, 95% CI: 1.3, 2.1), lack of availability of latrine (OR: 2.0, 95%CI: 1.3, 3.2), urban residence (OR: 1.9, 95%CI: 1.2, 3.0), and maternal hand washing (OR: 2.2, 95%CI: 2.0, 2.6) were significantly associated with childhood diarrhea.

**Conclusion:**

In this study, diarrhea among under-five children in Ethiopia was significantly high. Lack of maternal education, lack of availability of latrine, urban residence, and lack of maternal hand washing were significantly associated with childhood diarrhea.

## Introduction

Childhood diarrhea is defined as the passage of three or more loose or watery stools per 24 hours or an increase in stool frequency or liquidity that is considered abnormal by the mother [1, 2]. Despite remarkable progress in the reduction of under-five mortality, childhood diarrheal disease is still a leading cause of mortality and morbidity [3, 4]. Globally, diarrheal disease contributed to 15% of all under-five deaths (approximately 2.5 million deaths each year), making diarrheal disease the second leading cause of death in the youngest members of society [5, 6]. Developing countries or economically disadvantaged regions carried the highest burden of under-five mortality, with nearly four fifths of all under-five mortality occurring in Sub-Saharan Africa and south Asia [7, 8]. According to World Health Organization (WHO) (2016), the under-five mortality rate in low-income countries was 73.1 deaths per 1000 live births, nearly 14 times the average rate in high-income countries (i.e., 5.3 deaths per 1000 live births) [9].

In Ethiopia, diarrheal diseases are major contributors to under-five mortality. According to the 2016 Ethiopia Demographic and Health Survey report,12% of under-five children had a diarrheal episode in the 2 weeks before the survey [10]. More than half of under-five child deaths are attributable to diseases that are easily preventable and treatable through simple, cost effective, and affordable interventions. Strengthening health systems to provide such interventions to all children will potentially save many young lives [7]. In 2015, the United Nation adopted the Sustainable Development Goals (SDGs) to reduce child mortality and to promote well-being for all children. The SDG goal #3 Target 3.2 aims to end preventable deaths of newborns and under-five children by 2030 [7]. Likewise, the Ethiopian government also implemented various strategies, such as the Health Extension Program, to prevent and control infectious diseases like diarrhea [11].

Despite interventions and innovations by a range of stakeholders, under-five mortality related to diarrhea remains a major concern, especially in developing countries like Ethiopia. In Ethiopia, several studies were conducted to estimate the prevalence as well as to identify modifiable factors of under-five diarrheal diseases [11–32]. However, the prevalence reflected in these small and fragmented studies varied widely and remained inconclusive. Besides prevalence, identifying modifiable risk factors is a critical step in identifying potential interventions. The lack of a nationwide study that determines the prevalence and determinants of diarrhea among under-five children is a significant gap. Therefore, this systematic review and meta-analysis aimed to determine the pooled prevalence and determinants of diarrhea among under-five years of children using available studies in Ethiopia. The findings from this systematic review will highlight the prevalence and determinants of childhood diarrhea with implications to improve health workers’ interventions, to ensure cost-effectiveness, and to accelerate the reduction of childhood diarrhea in Ethiopia.

## Methods

### Study design and setting

A systematic review and meta-analysis was conducted to estimate the prevalence and determinants of diarrhea among under-five children in Ethiopia. Ethiopia is located in the horn of Africa. It is bounded by Eritrea to the north, Djibouti and Somalia to the east, Sudan and South Sudan to the west, and Kenya to the south. Currently, the Ethiopian population is estimated to be 106,059,710 with 20.2% living in urban areas [33, 34].

### Search strategies

We prepared and presented this meta-analysis according to the Preferred Reporting Items for Systematic Reviews and Meta-Analysis (PRISMA) [35] (S1 Table). To find potentially relevant articles, a comprehensive search with no date limits was performed in the following databases: PubMed/MEDLINE, Web of Science, EMBASE, CINAHL, Google Scholar, Science Direct and Cochrane Library (Table 1). All searches were limited to articles written in English given that such language restriction does not alter the outcome of the systematic reviews and meta-analyses[36]. Gray literature of observational studies was searched through the review of reference lists and input of content experts. In addition, to find unpublished papers relevant to this systematic review and meta-analysis, some research centers, including the Addis Ababa Digital Library were searched. Studies identified by our search strategy were retrieved and managed using Endnote X7 (Thomson Reuters, Philadelphia, PA, USA) software. The search of the literature was conducted between the 1^st^ of October to the 1^st^ of November,2017. All papers published until the 1^st^ of November, 2017 were considered. The search used the following keywords “prevalence”, “diarrhea”, “diarrheoa”, “under-five”, “children”, “determinants”, “associated factors” and “Ethiopia”. The search terms were used separately and in combination using Boolean operators like “OR” or “AND”.

**Table 1:** Example of searches for the MEDLINE/ PubMed and Google Scholar databases to assess the prevalence and determinants of diarrhea among under-five children in Ethiopia.

Databases	Searching terms	Number of studies
**Google scholar**	"prevalence" and "determinants" or "associated factors " and "children" or "under-five" and "diarrhea" or "diarrhoea" and "Ethiopia"-Adults	**124**
**MEDLINE/ PubMed**	("epidemiology"[Subheading] OR "epidemiology"[All Fields] OR "prevalence"[All Fields] OR "prevalence"[MeSH Terms]) AND ("diarrhoea"[All Fields] OR "diarrhea"[MeSH Terms] OR "diarrhea"[All Fields]) AND under-five[All Fields] AND ("child"[MeSH Terms] OR "child"[All Fields] OR "children"[All Fields]) AND ("ethiopia"[MeSH Terms] OR "ethiopia"[All Fields])	**43**
**From other databases**		**368**
**Total retrieved articles**		**535**
**Final full text relevant to our review**		**31**

### Eligibility criteria

#### Inclusion criteria

**Study area**: Only studies conducted in Ethiopia

**Population:** Only studies involving under-five year children

**Publication condition:** Both published and unpublished articles were included

**Study design:** All observational study designs (i.e., cross-sectional, case-control and cohort) reporting the prevalence of diarrhea in under-five children were eligible for this review.

**Language:** Only articles reported in English language were considered

#### Exclusion criteria

Articles, which were not fully accessible, after at least two-email contact with the primary authors, were excluded. Exclusion of these articles is because of the inability to assess the quality of articles in the absence of full text.

### Measurement of outcome variables

This study has two main outcomes. Childhood diarrheal disease, as the primary outcome variable of this study, is defined as having three or more loose or watery stools in a 24 hour period [37, 38]. The prevalence of childhood diarrhea was estimated as the total number of diarrhea cases divided by the total number of under-five children participating in the study multiplied by 100. The second outcome of this study was to identify the determinants of diarrhea among under-five children. For the second outcome, we determined the association between under-five diarrhea and determinants in the form of the log odds ratio. For major determinants, the odds ratio was calculated based on binary outcomes from the primary studies. The determinants included in this review were: residence (urban versus rural), educational status of caregiver (unable to read and write versus able to read and write), hand washing practices of caregiver (yes versus no), and availability of any type of latrine (yes versus no).

### Data extraction

Data from included articles were extracted using a standardized data extraction format, adapted from the Joanna Briggs Institute (JBI), by two authors (AA and CT) independently extracting all necessary data. Any disagreements during the data extraction were resolved through discussion and consensus (i.e., a Delphi process). The primary author of the original research was contacted for additional information or to clarify method details as needed. For the first outcome (prevalence), the data extraction format included primary author, publication year, region(s) of the country where the study was conducted, study area, sample size, response rate and prevalence with 95%CI. For the second outcome (determinants), data were extracted in a format of two by two tables, and then the log odds ratio for each factor was calculated based on the findings of the original studies.

### Risk of bias

Two authors (AA and CT) independently assessed the risk of bias for each original study using the tool. To assess the risk of bias, we used the Hoy 2012 addressing internal and external validity tool using 10 criteria [39]. The tool mainly included (1) representation of the population, (2) sampling frame, (3) methods of participants’ selection, (4) non-response bias, (5) data collection directly from subjects, (6) acceptability of case definition, (7) reliability and validity of study tools, (8) mode of data collection, (9) length of prevalence period, and (10) appropriateness of numerator and denominator. Each item was classified as either low or high risk of bias. Not clear was classified as high risk of bias. Finally, the overall score of risk of bias was then categorized according to the number of high risk of bias per study: low (≤ 2), moderate (3–4), and high (≥ 5) (S2 Table).

### Data processing and analysis

Data were extracted in Microsoft Excel format, followed by analysis using STATA Version 13 statistical software. The standard error for each original study was calculated using the binomial distribution formula. Heterogeneity among reported prevalence was assessed by computing p-values of Cochrane Q-test and *I*^2^statics [40]. As the test statistic showed there is significant heterogeneity among the studies (*I*^2^ = 96.31%, p <0.001) as a result a random effects meta-analysis model was used to estimate the DerSimonian and Laird’s pooled effect. In the current meta-analysis, arcsine-transformed proportions were used. The pooled proportion was estimated by using the back-transform of the weighted mean of the transformed proportions, using arcsine variance weights for the fixed-effects model and DerSimonian-Laird weights for the random-effects model [41]. To minimize the random variations between the point estimates of the primary study subgroup, analysis was done based on study settings (i.e., region(s) where the study occurred). In addition, to identify the possible source of heterogeneity, univariate meta-regression was undertaken by considering year of publication, quality score, region of the country where the study was conducted and sample size, however, none of these were found to be statistically significant. Egger’s and Begg’s tests at 5% significant level were not significant for publication bias [42]. Point prevalence, as well as 95% confidence intervals, was presented in the forest plot format. In this plot, the size of each box indicated the weight of the study, while each crossed line refers to 95% confidence interval. For the second outcome, a log odds ratio was used to determine the association between determinant factors and diarrhea among under-five children in the included evidence set.

## Results

**Fig 1:**
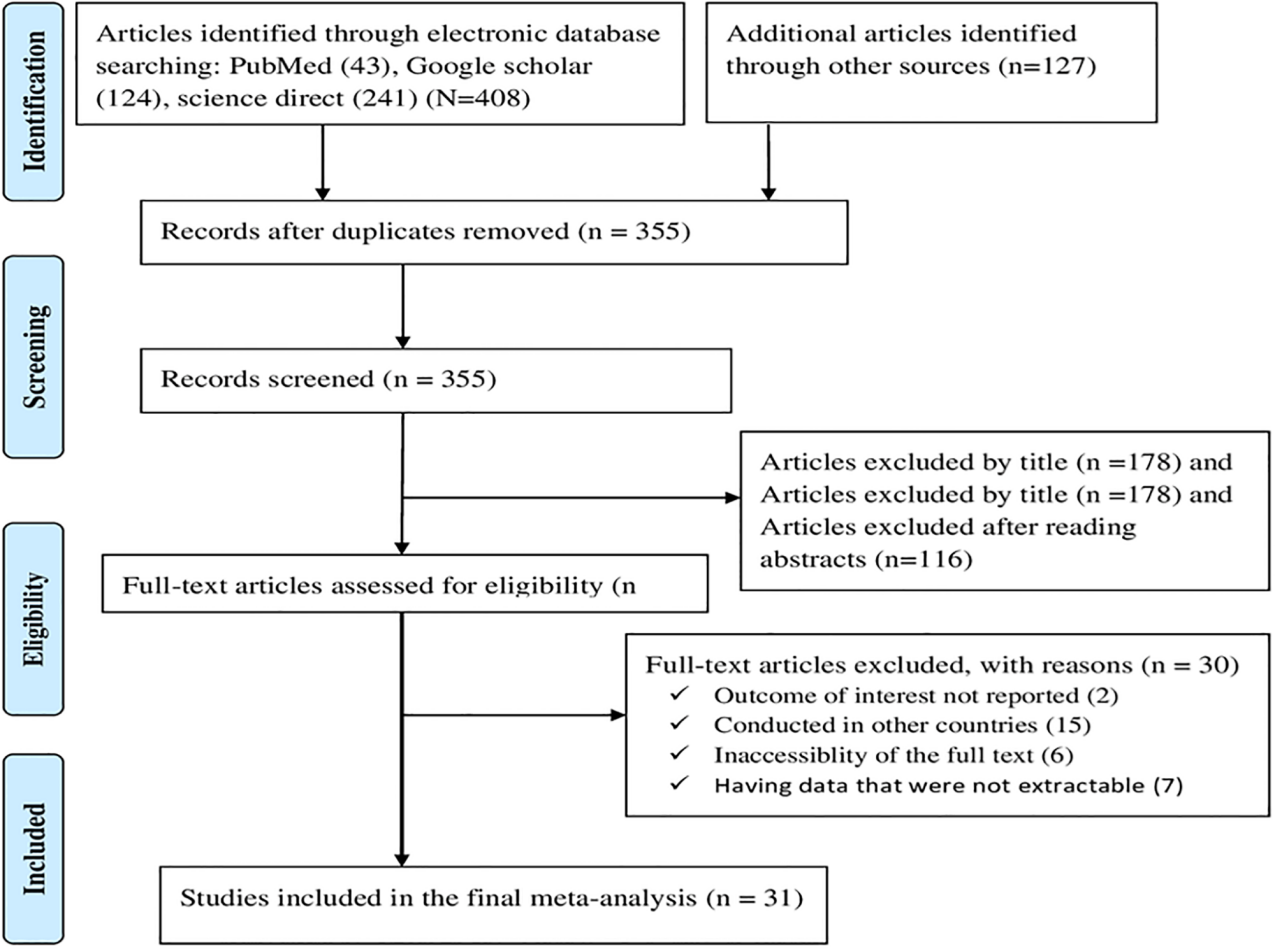
Flow chart of study selection for systematic review and meta-analysis of the prevalence and determinants of diarrhea among under-five children in Ethiopia.

Initially, 535 articles were retrieved reporting prevalence and determinants of diarrhea among under-five children using the range of databases previously described. Of these initial articles, 180 articles were excluded due to duplication. From the remaining 355 articles, 294 articles were excluded after review of their titles and abstracts confirmed non-relevance to this review. Therefore, 61 full text articles were accessed, and assessed for eligibility based on the pre-set criteria, which resulted in further exclusion of 30 articles primarily due to the study locations [30, 43–70] (S3 Table). Ultimately, 31 studies met the eligibility criteria and were included in the final meta-analysis (see Fig 1).

### Description of included studies

As described in Table 1, the 31included studies were cross-sectional study design, and published between 2003 to 2017. In the current meta-analysis, 22,744 study participants were involved to determine the pooled prevalence of diarrhea among under-five children. Regarding sample size, the sample size of the studies ranged from 278 to1, 807. The lowest prevalence (8%) of under-five diarrhea was reported in studies conducted in Wolitta Soddo Town, Southern Nations, Nationalities, and Peoples Region (SNNPR) [32] and Mecha District, Amhara region [28]whereas the highest prevalence (37%) was reported in a study conducted in a rural Dire Dawa [18]. In the present meta-analysis, six Ethiopian regions and two administrative towns were represented. Ten of the studies were from Amhara [19, 24, 25, 28, 31, 71–75], five from SNNPR [20, 26, 32, 76, 77], five from Oromia [17, 18, 21, 22, 78], three from Afar [13, 79, 80], three from Addis Ababa [12, 15, 29], two from Tigray [14, 81], two from Somali [23, 82] and one from Dire Dawa [18]. No studies were reported from Benishangul Gumiz, Harari, and Gagmbela regions. Regarding to response rate, almost all studies had a good response rate (>85%), which may, in part be attributable to the use of interviewer-administered questionnaires to collect the data (Table 2).

**Table 2:** Descriptive summary of 31 studies included in the meta-analysis of the prevalence and determinants of diarrhea among under-five children in Ethiopia 2017.

No	Author	Publication Year	Region	Study Area	Sample Size	Response Rate	Prevalence with 95%
1.	Hailemariam Berhe[81]	2016	Tigra	Enderta	278	94.2	36 (30, 41)
2.	Mohammed et al [20]	2013	SNNPR	Arba Minch	590	100	31 (27, 34)
3.	Mohammed and Zungu[21]	2016	Oromia	Sebeta	477	100	10 (7, 13)
4.	Mengistie et al [22]	2013	Oromia	Kersa District	1456	97.8	16 (14, 18)
5.	Woldu et al [13]	2016	Afar	Hadaleala District	704	100	26 (23, 30)
6.	Regassa and Lemma [16]	2016	Oromia	Adama Rural District	442	100	15 (12, 18)
7.	Teklit Angesom[14]	2015	Tigray	Laelay-Maychew District	543	100	18 (15, 21)
8.	Dessalegn et al [28]	2011	Amhara	Mecha District	768	100	8 (6, 10)
9.	Gedamu et al [25]	2017	Amhara	FartaWereda	988	99	17 (15, 19)
10.	Zeleke and Alemu [12]	2014	Addis Ababa	Yeka Sub City	350	100	33 (29, 39)
11.	Anteneh et al [31]	2017	Amhara	Jabithennan District	775	99.2	25 (22, 28)
12.	Gedefaw et al [24]	2015	Amhara	Bahir Dar	667	99.1	22 (19, 25)
13.	Tadesse Yared[15]	2016	Addis Ababa	Yeka Sub City Woreda 3	399	99.7	9 (6, 11)
14.	Hashi et al [23]	2016	Somalia	Jigjiga District	1807	100	27 (25, 29)
15.	Alambo Kedir Addisu[32]	2015	SNNPR	Wolitta Soddo Town	954	98.4	8 (6, 10)
16.	Regassa et al [17]	2008	Oromia	Nekemte Town	461	96.6	29 (25, 33)
17.	Demelash Ayele[29]	2014	Addis Ababa	Addis Ababa	348	83.3	30 (25, 35)
18.	Jamboree et al [26]	2016	SNNPR	Gummer Woreda, Guragie	611	96.4	15 (12, 18)
19.	Mulugeta Teklu[19]	2003	Amhara	Meskanena Mareko Woreda	987	100	22 (20, 25.0)
20.	Keneni et al [18]	2016	Dire Dawa	Dire Dawa Rural District	291	98.6	37 (32, 43)
21.	Gebru et al [77]	2014	SNNPR	Sheko Rural District	792	96	19 (17, 22)
22.	Awoke [72]	2013	Amhara	Bahir Dar Town	415	98.34	27 (22, 31)
23.	Tamiso et al [76]	2014	SNNPR	rural area of Shebedino	769	98.8	20 (17, 23)
24.	Mamo and Hailu [71]	2014	Amhara	Debre Birehan Town	483	100	32 (28, 36)
25.	Bitew et al [80]	2017	Afar	Hadaleala District	704	NR	26 (23, 30)
26.	Gizaw et al [79]	2017	Afar	Hadaleala District	367	100	31 (27, 36)
27.	Ayele et al [74]	2014	Amhara	Enemay District	634	100	19 (16, 22)
28.	Mekasha and Tesfahun[78]	2003	Oromia	Jimma Town	605	NR	23 (20, 27)
29.	Alelign et al [75]	2016	Amhara	Debre Birehan Town	312	NR	12 (9, 16)
30.	Getu et al [73]	2014	Amhara	Dejen District	710	96.5	24 (21, 27)
31.	Bizuneh et al [82]	2017	Somali	Jigjig Town	492	92.8	15 (12, 18)

NR: not reported, SNNPR: Southern Nations, Nationalities, and Peoples Region.

### Risk of bias

The risk of bias for each original study was conducted using a risk of bias tool which encompassed ten different items [39]. Among the 31 included studies, our summary assessment revealed that more than three fourth (77.4%) of the included studies had low risk of bias [12–14, 17–19, 22–26, 28, 29, 32, 71, 73–80, 82] whereas, about 16.1% of the included studies had moderate risk of bias [15, 16, 21, 31, 72] the remaining, 6.5% of the studies had high risk of bias [20, 81].

### Prevalence of diarrhea among under-five children in Ethiopia

**Fig 2:**
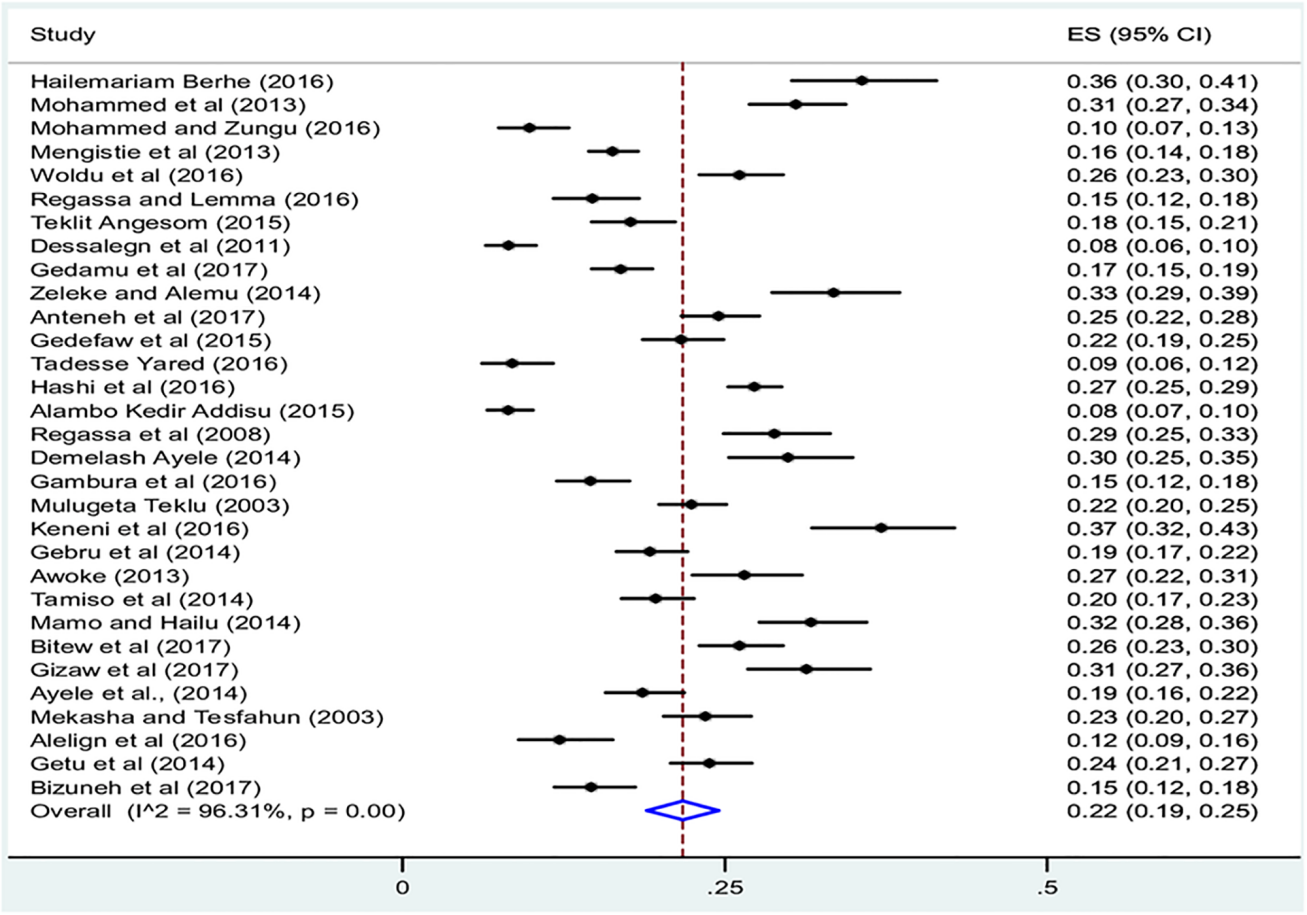
Forest plot of the pooled prevalence of diarrhea among under-five children in Ethiopia.

**Fig 3:**
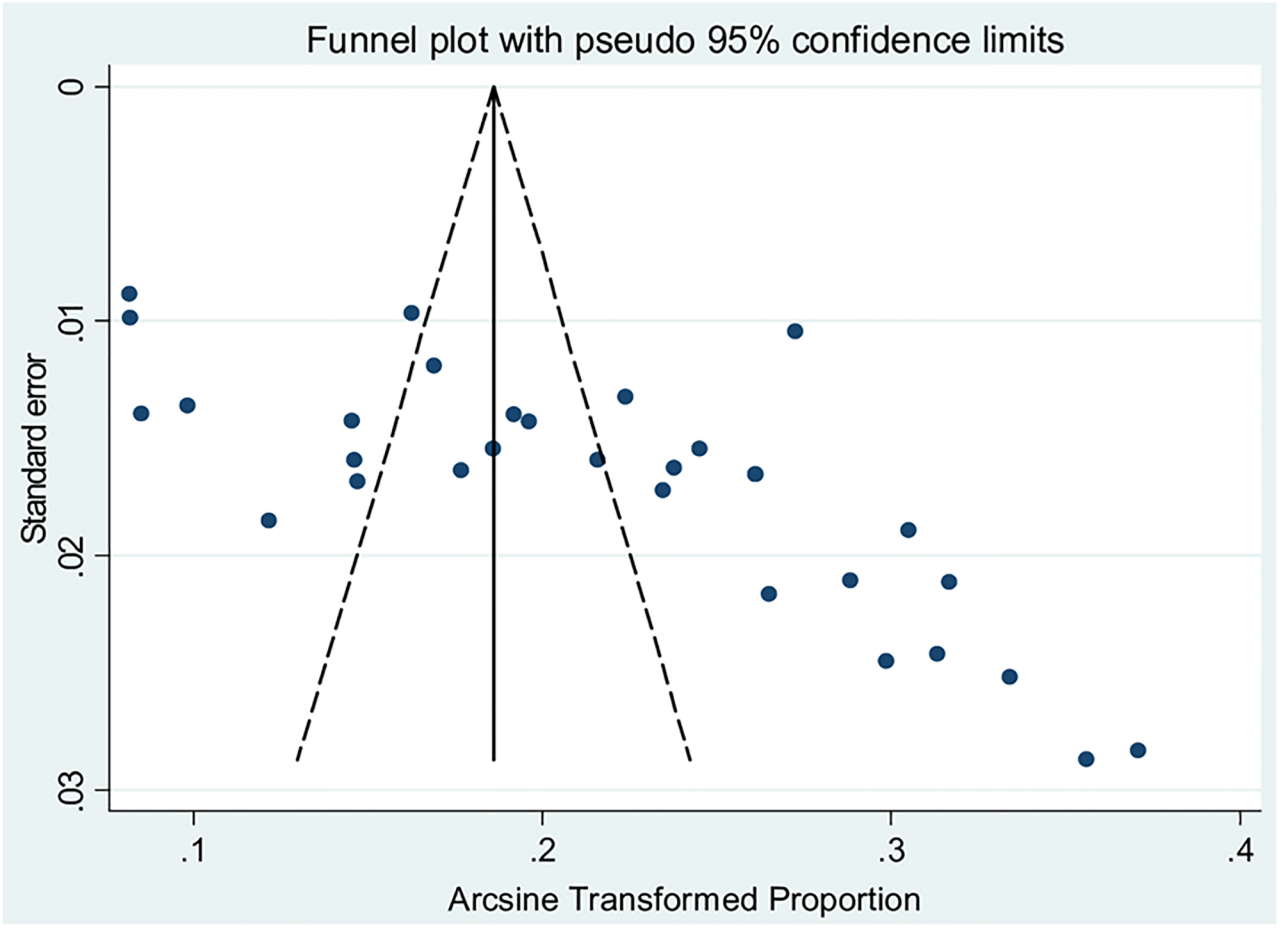
Funnel plot with 95% confidence limits of the pooled prevalence of diarrhea among under-five children in Ethiopia.

The 31 included studies revealed that a pooled prevalence of under-five diarrhea in Ethiopia was 22% (95%CI: 19, 25) (Fig 2). High heterogeneity was observed across the included studies (I^2^ = 96.31, p<0.001). Therefore, a random effect meta-analysis model was computed to estimate the pooled prevalence of under-five diarrhea in Ethiopia. From this meta-analysis, the highest prevalence was 37% (95%CI: 32, 43) reported in a study by Keneni et al [18]whereas the lowest prevalence of 8% was reported elsewhere [28, 32]. To identify possible sources of heterogeneity, different factors associated with heterogeneity, such as year of publication, quality score, region of the country where the study conducted and sample size, were investigated by using univariate meta-regression models, although none of these variables were found to be statistically significant (Table 3). Funnel plot asymmetry was used to check the presence of publication bias (Fig 3). The result of funnel plot showed that there was a slight asymmetrical distribution of articles. To confirm this asymmetry, we conducted an objective (Begg’s and Egger’s tests) based tests. The results of Begg’s and Egger’s tests showed that there was no statistical significant publication bias in estimating the prevalence of diarrhea among under-five children [(p = 0.2) and (p = 0.4) respectively].

**Table 3:** Related factors with heterogeneity of diarrheal prevalence among under-five children in the current meta-analysis (based on univariate meta-regression).

Variables	Coefficient	P-value
Publication year	-0.17	0.70
Sample size	-0.004	0.30
Quality score	0.006	0.98
**Regions**		
Addis Ababa	-2.44	0.72
Afar	1.73	0.80
Amhara	-5.51	0.33
Oromia	-7.53	0.23
SNNPR	-7.76	0.22
Tigray	0.22	0.97
Somali and Dire Dawa (Constant)	26.01	<0.001

### Subgroup analysis

In this meta-analysis, we performed subgroup analysis based on the region of the country where studies were conducted as well as sample size. Accordingly, the highest prevalence was observed in Afar region with a prevalence of 27% (95% CI: 25, 30) followed by Somali and Dire Dawa regions at 26% (95% CI: 15, 37) and then Addis Abeba at 24% (95% CI: 7, 41). With regard to sample size, the prevalence of diarrhea was higher in studies having a sample of size<670, 23% (95% CI: 19, 27) compared to those having a sample size> = 670, 20% (95%CI: 16, 24) (Table 4).

**Table 4:** Subgroup prevalence of diarrhea among under-five children in Ethiopia, 2017 (n = 31).

Variables	Characteristics	Included studies	Sample size	Prevalence with (95% CI)
**By region**	Amhara	10	6,714	21 (16, 25)
	Oromia	5	3,441	18 (13, 24)
	Afar	3	1,775	27 (25, 30)
	Addis Ababa	3	1,079	24 (7, 41)
	SNNPR	5	3,716	18 (11, 25)
	Tigray	2	821	22 (19, 25)
	Somali and Dire Dawa	3	2,590	26 (15, 37)
**By sample size**	> = 670	12	10,704	20 (16,24)
	<670	19	6,722	23 (19, 27)
**Overall**		31	22,744	22(19, 25)

### Determinants of under-five diarrhea in Ethiopia

#### The association between maternal education and childhood diarrhea

**Fig 4:**
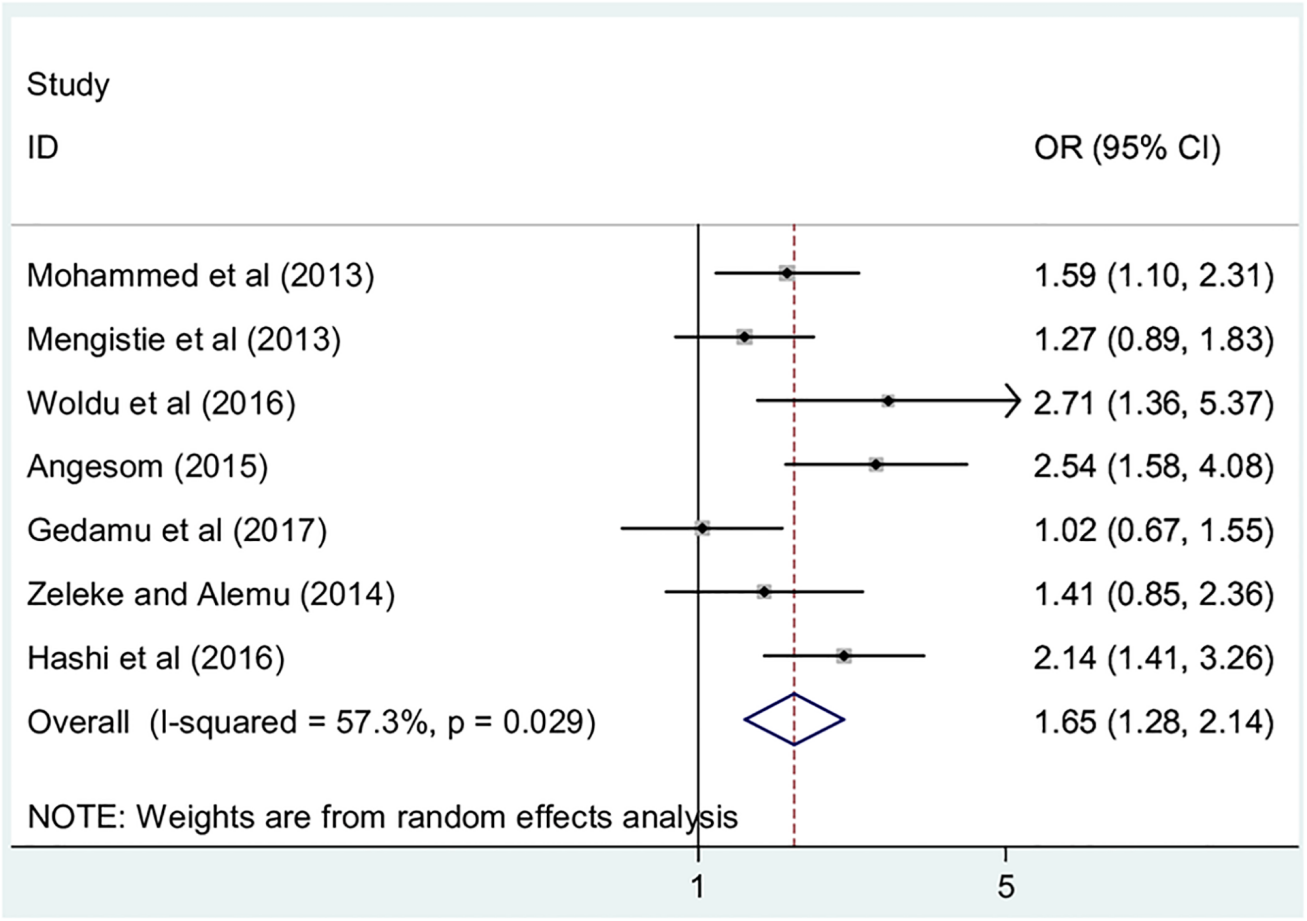
The pooled odds ratio of the association between maternal education and childhood diarrhea in Ethiopia.

In this meta-analysis, we examined association between maternal educational status and childhood diarrhea by using seven studies [12–14, 20, 22, 23, 25]. The findings from these seven studies revealed that the occurrence of childhood diarrhea was significantly associated with mothers’ educational status. Accordingly, the likelihood of diarrhea occurrence was 1.7 times higher among children whose mothers’ were unable to read and write as compared to their literate counterparts (OR: 2.5, 95% CI: 1.3, 2.1). The result of the test statistics indicated that moderate heterogeneity (I^2^ = 57.3% and p = 0.03) was presented across the included studies. Therefore, a random effect meta-analysis model was employed to determine the association(Fig 4).

#### Association between latrine availability and childhood diarrhea

**Fig 5:**
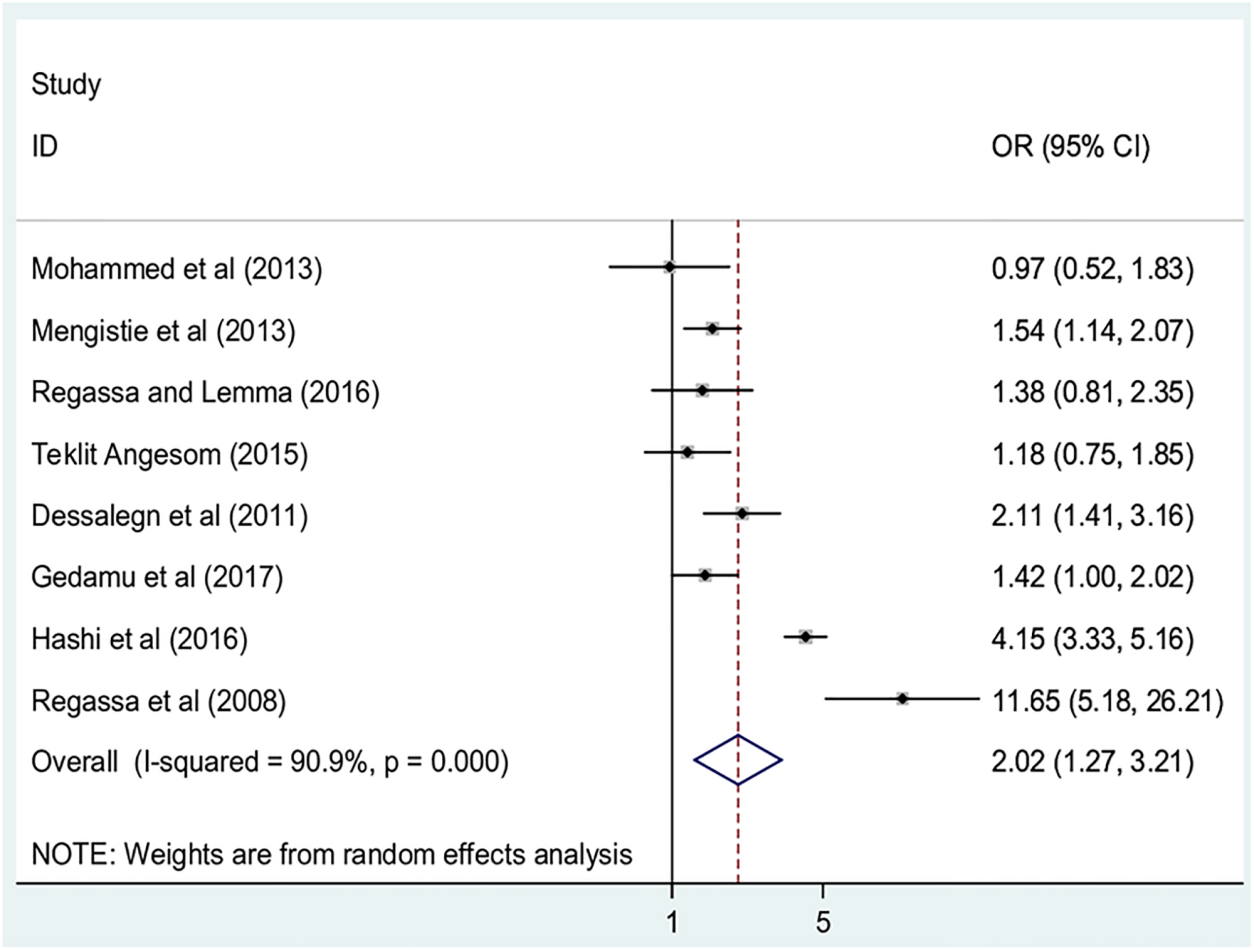
The pooled odds ratio of the association between latrine availability and childhood diarrhea in Ethiopia.

Similarly, the association between the availability of any type of latrine and childhood diarrhea was examined by using eight studies [14, 16, 17, 20, 22, 23, 25, 28]. Four of the included studies reported that the availability of any type of latrine were not significantly associated with childhood diarrhea [14, 16, 20, 25] whereas, four indicated that absence of any type of latrine was positively associated with childhood diarrhea [17, 22, 23, 28]. The result of this meta-analysis revealed that the absence of any type of latrine was positively associated with childhood diarrhea. From this result, children living in households without latrine facilities were found to be 2.0 times more likely to develop diarrhea than children living in households with such facilities (OR: 2.0, 95%; CI: 1.3, 3.2) (Fig 5). The included studies exhibited high heterogeneity (I^2^ = 90.9% and p< 0.001), hence random effect meta-analysis was computed.

#### Association between residence and childhood diarrhea

**Fig 6:**
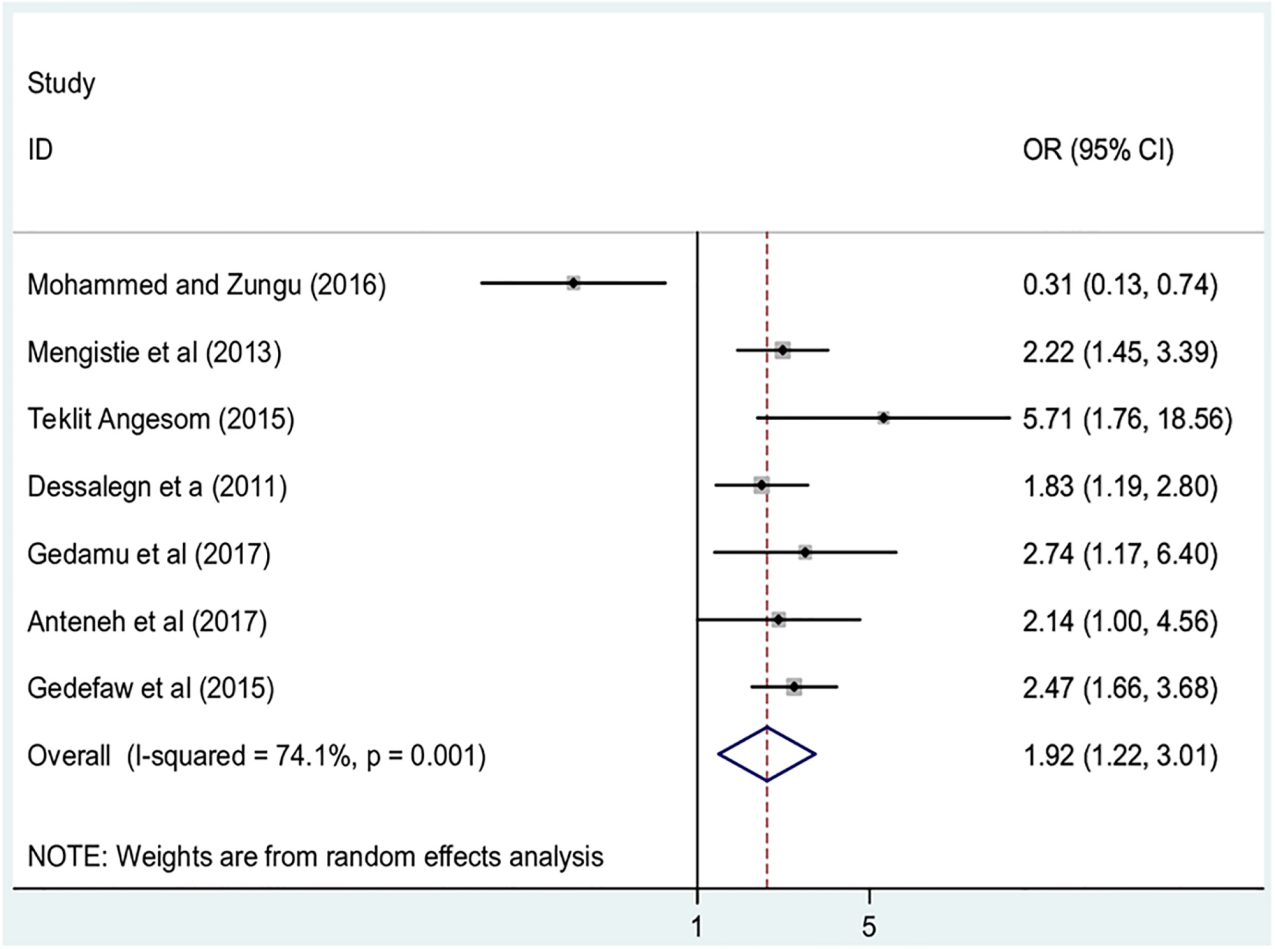
The pooled odds ratio of the association between residence and childhood diarrhea in Ethiopia.

To examine the association between residence and childhood diarrhea, studies that examined the association between respondents’ residences and under-five diarrhea were included [14, 21, 22, 24, 25, 28, 31]. According to Mohammed and Zungu [21], children from households of rural households were less likely to have diarrhea than their urban counterparts. Conversely, five studies disclosed that children from rural households were more likely to have diarrhea as compared to children from urban households [14, 22, 24, 25, 28]. One study [31]found residence was not significantly associated with childhood diarrhea. The pooled result of this meta-analysis indicated that children from rural households were 1.9 times more likely to have diarrhea as compared to their urban counterparts (OR: 1.9, 95%CI: 1.2, 3.0) (Fig 6). In this meta-analysis, included studies were characterized by high heterogeneity (I^2^ = 74.1%; p = 00.1), we computed a random effect meta-analysis.

#### Association between maternal hand washing practices and childhood diarrhea

**Fig 7:**
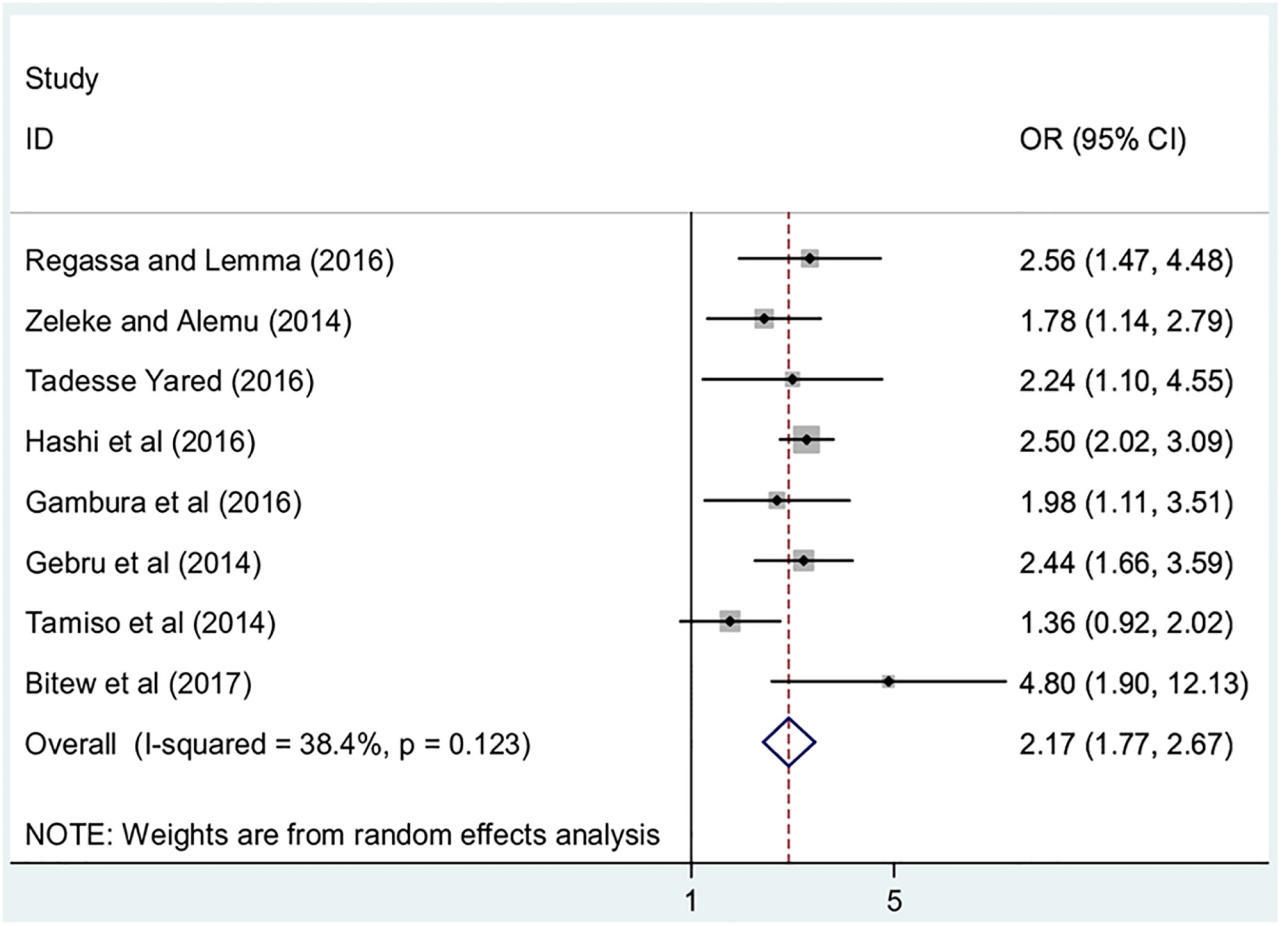
The pooled odds ratio of the association between maternal hand washing practices and childhood diarrhea in Ethiopia.

Finally, in this review, we examined the association between mothers’ hand washing practices and childhood diarrhea. Eight studies that examined the association between childhood diarrhea and mothers hand washing practice were included. Seven studies indicated that mothers’ hand washing practice after toilet visiting was significantly associated childhood diarrhea [12, 15, 16, 23, 26, 80]. These studies reported that mothers who did not practice hand washing after visiting a toilet were positively associated with childhood diarrhea. One study reported that mothers’ hand washing practices were not significantly associated with diarrhea [76]. The overall result of this study revealed that children whose mothers did not practice hand washing after visiting a toilet were 2.3 more likely to develop diarrhea as compared to their counterparts (OR: 2.2, 95%CI: 2.0, 2.6) (Fig 7). Moderate heterogeneity (I^2^ = 38.4%; p-value = 0.12) was observed among the included studies; hence, a random effect meta-analysis model was employed to estimate the final analysis.

## Discussion

Diarrhea is one of the major causes of morbidity and mortality among under-five children in Ethiopia. Based on the WHO estimates, diarrhea contributes to more than one in every ten (13%) child deaths in Ethiopia [83]. Estimating the pooled prevalence of under-five diarrhea and its contributing factors in Ethiopia may contribute to informing policy makers. To the best our knowledge, this meta-analysis is the first of its kind to estimate the pooled prevalence of diarrhea and its determinants among under-five children in Ethiopia.

The overall prevalence of under-five diarrhea obtained from this study indicated that almost one in five (22%; 95% CI: 19, 25) children under the age of five in Ethiopia experienced diarrhea. The result of this meta-analysis is in line with the 2000 Ethiopian DHS [84, 85] report, which shows the prevalence of diarrhea as 24%. However, this finding is almost two times higher than the 2011 Ethiopian DHS, which suggests 13% of under-five children had diarrhea [86]. In addition, this finding is much higher than 2016 Ethiopian DHS [10]and 2005 [87] reports with reported prevalence of childhood diarrhea of 12% and 18% respectively. Similarly, our finding is two times higher than Ghana’s 2014 DHS (12%) [88], higher than Kenya’s 2014 DHS finding of 15% [89]. The possible explanation for the above variation could be attributed to methodological variation in the assessment of prevalence. The difference in the prevalence of diarrhea between our study and other sub-Saharan countries could be explained by the difference in socio-demographics and sociocultural practices, which has a great impact on child feeding.

The subgroup analysis of this study indicated that the highest prevalence of diarrhea was observed in Afar region, 27% (95% CI: 25, 30) followed by Somali and Dire Dawa regions, 26% (95% CI: 15, 37) whereas the lowest prevalence was observed in Oromia and SNNPR with prevalences of 18% (95%CI: 13, 24) and 18% (95%CI: 11, 25) respectively. This finding of this study is in agreement with the 2011 Ethiopian DHS, which shows Somali region (19.5%) has higher prevalence of diarrhea next to Benishangul-Gumuz (22.7%) and Gambela (22.6%) regions [86]. The possible explanations for this variation might be due to the difference in basic environmental and behavioral characteristics of caregivers. Another possible explanation for this variation could be due to the difference in the socio-demographic, environmental, and behavioral characteristics of households. As the communities living in Somali and Afar regions were nomadic, they go from place to place in search of pasture and water. They have no permanent residential places, hence, lacking access to basic healthcare facilities and sanitation services. The main sources of water for these populations were rivers, streams, and wells that are high risk for contamination. In addition, those populations more commonly practicing open defecation, which leads to the living environment, is polluted with human excreta that are the main source of diarrheal disease, especially for the children who routinely play in the unhygienic environment. Moreover, people suffering from illiteracy and poverty, which, in turn, deteriorates their quality of life. All these phenomena are the direct risk factors for the occurrence of childhood diarrheal disease [90]

The present study was also aimed to identify the determinants of diarrhea among under-five children in Ethiopia. In this study, lack of maternal education, lack of availability of latrine, urban residence, and lack of maternal hand washing were significantly associated with childhood diarrhea. The likelihood of diarrhea occurrence was 1.7 times higher among children whose mothers were unable to read and write as compared to their counterparts. The finding of this study is consistent with studies done in Ghana and Nigeria, which showed that the prevalence of diarrhea was significantly, varieties in related to the caregiver’s educational status. These studies reported that diarrhea was higher among children whose mothers have no formal education [91–93]. This finding may relate to these educated mothers have better knowledge about the rules of hygiene, appropriate child feeding practices, and early signs and symptoms of diarrhea which are the major determinant factors for the occurrence of childhood diarrhea. In addition, education has a great impact in changing behaviors at the household level. Moreover, education may increase the mother’s awareness about methods of transmission and prevention of diarrhea.

Latrine availability was another determinant of under-five diarrhea. Accordingly, children living in households without latrine facilities were 2.0 times more likely to develop diarrhea than children living in households with such facilities. This finding is congruent with a study conducted in Tanzania [94]. Different studies also reported that the absence of latrine facility was strongly associated with the occurrence of diarrheal disease [69, 70, 95]. The accessibility to a latrine in the family unit is an indication of sanitation conditions, which will have an implications to prevent the possibility of transmission of pathogens through fecal contamination [96].

Furthermore, it is indicated that children living in rural areas were more vulnerable to diarrhea than their urban counterparts. Children from rural households were 1.9 times more likely to have diarrhea as compared to their counterparts. This finding is contradictory to a study reported from Iraq [97]; however, similar to a study conducted in Pakistan [98]. This variance could be due to the population living in urban areas having better access to an improved water source, sanitation facility, health care facility and better knowledge about the prevention and control of diarrheal disease in comparison to rural populations. Another possible reason could be that people living in rural areas tend to be poorer than their urban counterparts are, a factor known to have an impact on the level of hygienic practice.

Lastly, lack of maternal hand washing was significantly associated with childhood diarrhea. Children whose mothers did not practice hand washing after visiting a latrine were 2.3 more likely to develop diarrhea as compared to their counterparts. This finding to those of a study conducted in Nigeria [91]. The rationale for hand washing after going to latrine to reduce the load of microorganisms has been well documented as fecal-oral microorganism transmissions due to post-defecation contamination of hands and fingers is well known [20, 22, 77, 99]. As mothers are the most frequent primary caregivers for their children, it is important to assess the contribution of maternal hand washing practices.

## Limitations of the study

This meta-analysis has several limitations. The first limitation of this study was only English articles or reports were considered to conduct this nationally based review. In addition, all of the studies included in this review were cross-sectional in nature; as a result, the outcome variable might be affected by other confounding variables. The majority of the studies included in this review had a relatively small sample size, which could affect the estimated prevalence reported. Almost all research included in this meta-analysis used the WHO tool for diarrheal assessment, its occurrence was determined based on the reports of mothers without the confirmation of physicians. Therefore, this result might be affected social desirability bias. Furthermore, this meta-analysis represented only studies reported from six regions and two administrative town of the country, which may reflect under-representation due to the limited number of studies included.

## Conclusion

In this study, diarrheal disease among under-five children in Ethiopia was significantly high. In addition, childhood diarrhea is significantly higher in nomadic population. Lack of maternal education, lack of availability of latrine, urban residence, and lack of maternal hand washing found significantly associated with childhood diarrhea. Therefore, based our findings, we recommend particular emphasis shall be given to the rural communities. Moreover, health educations about personal hygiene as well as, proper disposal of wastes including excreta in integration with the existing national health extension program are recommended.

## Supporting information

S1 Table PRISMA checklist. (DOC)

S2 Table Risk of bias assessment of included studies. (XLSX)

S3 Table List of excluded full texts and reasons of exclusion. (DOCX)

## Abbreviations

CI: Confidence Interval
DHS: Demographic and Health Survey
EDHS: Ethiopia Demographic and Health Survey
OR: Odds ratio
SDG: Sustainable Development Goals
SNNPR: Southern Nations, Nationalities, and Peoples Region
WHO: World Health Organization
