# Supplementary material for: Prevalence and determinants of diarrhea among under-five children in Ethiopia: A systematic review and meta-analysis
Source: PLoS One. 2018 Jun 28;13(6):e0199684. doi: 10.1371/journal.pone.0199684 (PMC6023116; doi:10.1371/journal.pone.0199684)
Supplement: S3 Table — (DOCX) [file pone.0199684.s003.docx]

**Additional file 1. List of excluded references and reasons for exclusion.**

| **References** | **Reasons** |
| --- | --- |
| 1. Warsame HA, Chomi E, Ngwatu P: **Influence of Health System Factors on Morbidity of Diarrheal Disease among Under Five Years Children Living in Internally Displaced Population Camps of Hodan District, Mogadhishu-Somalia**. *The Journal of Global Health Care Systems* 2016, **6**(2). | 1 |
| 1. Uwizeye D, Sokoni CH, Kabiru CW: **Prevalence and correlates for diarrhoea in the mountainous** **informal settlements of Huye town, Rwanda**. *SpringerPlus* 2014, **3**(1):745. | 1 |
| 1. Tambe AB, Nzefa LD, Nicoline NA: **Childhood Diarrhea Determinants in Sub-Saharan Africa: A Cross Sectional Study of Tiko-Cameroon**. *Challenges* 2015, **6**(2):229-243. | 1 |
| 1. Siziya S, Muula A, Rudatsikira E: **Correlates of diarrhoea among children below the age of 5 years in Sudan**. *Afr Health Sci* 2013, **13**(2):376-383. | 1 |
| 1. Sayed YN, El-Sayed AZM, El-Fattah SAA: **Effect of socioeconomic status on infectious diarrhea in Egyptian children** | 1 |
| 1. Osman F, Abdirisaq H, Abdirahman M, Sayid M: **Factors Influencing the Occurrence of Diarrhoea Among Children Under the Age of Five Admitted to Benadir Hospital Mogadishu-Somalia**. *JAMHURIYA UNIVERSITY*:1. | 1 |
| 1. Mulatu G, Beyene G, Zeynudin A: **Prevalence of Shigella, Salmonella and Campylobacter species and their susceptibility patters among under five children with diarrhea in Hawassa town, south Ethiopia**. *Ethiop J Health Sci* 2014, **24**(2):101-108. | 3 |
| 1. Kelly P, Khanfir H, David PH, Arata M, Kleinau EF: **Environmental and behavioral risk factors for diarrheal diseases in childhood: a survey in two towns in Morocco**. In: *EHP Applied Study. Volume 79*, edn.: EHP; 1999. | 1 |
| 1. Karambu S, Matiru V, Kiptoo M, Oundo J: **Characterization and factors associated with diarrhoeal diseases caused by enteric bacterial pathogens among children aged five years and below attending Igembe District Hospital, Kenya**. *Pan Afr Med J* 2013, **16**:37. | 1 |
| 1. ELMI O, DIOSO RIP: **Prevalence of Diarrhoeal Diseases Among Children under Five Years in East African Countries from 2012–2017**. *ASEAN Journal on Science and Technology for Development* 2017, **34**(1):51-55. | 1 |
| 1. Debajyoti M, Panza A: **Demographic Socio-Economic and Environmental Factors Associated with Diarrhoea Morbidity in Children Under-Five in Rural Odisha: A Study of Rayagada District India**. *Journal of Health Research* 2013, **27**(6):375-381. | 1 |
| 1. Danquah L, Mensah CM, Agyemang S, Awuah E: **Risk factors associated with diarrhea morbidity among children younger than five years in the Atwima Nwabiagya District, Ghana: a cross-sectional study**. *Sci J Pub Health* 2015, **3**(3):344-352. | 1 |
| 1. Carvajal-Velez L, Amouzou A, Perin J, Maiga A, Tarekegn H, Akinyemi A, Shiferaw S, Young M, Bryce J, Newby H: **Diarrhea management in children under five in sub-Saharan Africa: does the source of care matter? A Countdown analysis**. *BMC Public Health* 2016, **16**:830. | 1 |
| 1. Bogale GG, Gelaye KA, Degefie DT, Gelaw YA: **Spatial patterns of childhood diarrhea in Ethiopia: data from Ethiopian demographic and health surveys (2000, 2005, and 2011)**. *BMC Infect Dis* 2017, **17**(1):426. | 3 |
| 1. Azage M, Haile D: **Factors affecting healthcare service utilization of mothers who had children with diarrhea in Ethiopia: evidence from a population based national survey**. *Rural and remote health* 2015, **15**(3493). | 3 |
| 1. Mitike G: **Prevalence of acute and persistent diarrhoea in north Gondar zone, Ethiopia**. *East Afr Med J* 2001, **78**(8):433-438. | 2 |
| 1. Mekasha A, Tesfahun A: **Determinants of diarrhoeal diseases: a community based study in urban south western Ethiopia**. *East Afr Med J* 2003, **80**(2):77-82. | 2 |
| 1. Teklemariam S, Getaneh T, Bekele F: **Environmental determinants of diarrheal morbidity in under-five children, Keffa-Sheka zone, south west Ethiopia**. *Ethiop Med J* 2000, **38**(1):27-34. | 2 |
| 1. Olango P, Aboud F: **Determinants of mothers' treatment of diarrhea in rural Ethiopia**. *Soc Sci Med* 1990, **31**(11):1245-1249. | 2 |
| 1. Berhe F, Berhane Y: **Under five diarrhea among model household and non model households in Hawassa, South Ethiopia: a comparative cross-sectional community based survey**. *BMC Public Health* 2014, **14**(1):187. | 3 |
| 1. Mengistie B, Berhane Y, Worku A: **Household water chlorination reduces incidence of diarrhea among under-five children in rural Ethiopia: a cluster randomized controlled trial**. *PLoS One* 2013, **8**(10):e77887. | 3 |
| 1. Lengerh A, Moges F, Unakal C, Anagaw B: **Prevalence, associated risk factors and antimicrobial susceptibility pattern of Campylobacter species among under five diarrheic children at Gondar University Hospital, Northwest Ethiopia**. *BMC Pediatr* 2013, **13**(1):82. | 3 |
| 1. Mediratta RP, Feleke A, Moulton LH, Yifru S, Sack RB: **Risk factors and case management of acute diarrhoea in North Gondar Zone, Ethiopia**. *Journal of health, population, and nutrition* 2010, **28**(3):253. | 1 |
| 1. Godana W, Mengiste B: **Environmental factors associated with acute diarrhea among children under five years of age in derashe district, Southern Ethiopia**. *Sci J Public Health* 2013, **1**(3):119-124. | 4 |
| 1. Tesfaye F, Enquselassie F, Kebede F, Wendimagegn G: **Usage of oral rehydration salt in acute childhood diarrhoea, Adami-Tullu Woreda**. *Ethiop Med J* 1996, **34**(3):163-171. | 2 |
| 1. Eshete WB: **A stepwise regression analysis on under-five diarrhoael morbidity prevalence in Nekemte town, western Ethiopia: maternal care giving and hygiene behavioral determinants**. 2008. | 4 |
| 1. Ketsela T, Asfaw M, Belachew C: **Knowledge and practice of mothers/care-takers towards diarrhoea and its treatment in rural communities in Ethiopia**. *Ethiop Med J* 1991, **29**(4):213-224. | 4 |
| 1. Kakulu RK: **Diarrhoea among under-five children and household water treatment and safe storage factors in Mkuranga district, Tanzania**. Muhimbili University of Health and Allied Sciences; 2012. | 1 |
| 1. Getaneh T, Assefa A, Tadesse Z: **Diarrhoea morbidity in an urban area of southwest Ethiopia**. *East Afr Med J* 1997, **74**(8):491-494. | 2 |
| 1. Mock NB, Sellers TA, Abdoh AA, Franklin RR: **Socioeconomic, environmental, demographic and behavioral factors associated with occurrence of diarrhea in young children in the Republic of Congo**. *Soc Sci Med* 1993, **36**(6):807-816. | 1 |

Reasons for exclusion: 1. Conducted in other countries; 2. Unable to find the full texts 3. Having data that were not extractable 4. The outcome of interests was not reported
